# Millennial Evolution of a Karst Socio-Ecological System: A Case Study of Guizhou Province, Southwest China

**DOI:** 10.3390/ijerph192215151

**Published:** 2022-11-17

**Authors:** Yetong Li, Qihua Ke, Zhuodong Zhang

**Affiliations:** 1 Faculty of Geographical Science, Beijing Normal University, Beijing 100875, China; 2 State Key Laboratory of Earth Surface Processes and Resource Ecology, Beijing Normal University, Beijing 100875, China

**Keywords:** millennial scale, socio-ecological system, land-use change, karst rocky desertification, soil erosion

## Abstract

The dynamic changes in socio-ecological system (SES) have exerted increasing pressures on the natural environment, leading to observable changes in terrestrial surface structure. Therefore, understanding the historical evolution mechanism of social ecosystems is crucial for the future sustainable management of karst regions. However, detailed quantitative analyses of karst socio-ecological system at a long-term scale are lacking. Here, we applied a comprehensive research framework for the SES of karst region to visually analyze the evolution of karst SES over the past 1000 years in Guizhou Province, defining five evolution stages of the karst SES. Concurrently, we characterized the interactive effects of drivers on karst socio-ecological system during every evolutionary stage, and then assess major influences between these stages. Despite rocky desertification as the main effect of karst SES driven by many indicators, the quantitative analysis indicated that human-dominated land-use change explained the expansion of rocky desertification. Although effective implementation of relevant policies partly compensated for increased environmental pressures, continued structure and function shifts in local ecosystem can challenge progress towards sustainability in karst region. Our findings provide scientific references for managers and policymakers to assist them to identify how environmental issues emerged in karst areas and how they should be addressed.

## 1. Introduction

Human land-use practices have been greatly modified the natural environment over the course of the Holocene, with up to 50% of the Earth’s ice-free surface having changed [1,2]. The changes in land and ecosystems and their impact on global environmental change and sustainability are major research challenges for human environmental science [3,4,5]. Historically, agriculture was one of the most important forms of land use and the main driver of global change [6]. The expansion of cropland driven by human has reshaped the surface landscape and has a direct and far-reaching impact on terrestrial ecosystems [7]. The rocky desertification is the most serious ecological and environmental issue in Southwest China [8,9]. Karst rocky desertification refers to the evolution of surface vegetation and soil in karst areas into rocky landscapes with almost no vegetation and soil [10]. Less flat land, thin soil layer, poor fertility, and serious soil erosion are the main challenges to the development of agriculture in karst areas [11,12,13,14,15]. Guizhou Province is the most serious rocky desertification in China, suffering serious ecological and environmental problems. It has experienced significant biophysical and socioeconomic changes over the past millennium. The expansion of population and cropland in this region severely restrict the sustainable development of the regional economy, due to the fragile karst ecosystem, prominent conflicts between man and land, serious soil erosion, and rocky desertification [16,17]. Therefore, understanding the historical evolution mechanism of social ecosystems is crucial for the future sustainable management of karst regions.

Recent studies indicated that the growing population exerted considerable pressure on the terrestrial surface ecological system of karst region for the last thousand years [18,19]. However, the evolution of karst region had never been quantified and analyzed on a long-term scale due to the lack of detailed historical data. Moreover, the current issues of historical evolution related to human–nature interactions in the Guizhou Province were reported in the form of narrative [20,21]. A detailed characterization of human–nature interactions can improve acknowledge of tensions between human activities and natural ecosystems, provide basis on ecological conservation of karst area, and help to understand regional sustainable development processes. Unfortunately, human behavior on structure and function of the karst ecosystem are unknown. Yet, it remains unclear how such human-driven changes in terrestrial surface ecological process have affected the sustainability of karst ecological system over time. Therefore, we need to efforts to quantify changes in indictors of nature and social systems to better understand the dynamics, drivers, and impacts of historical evolution. 

The emergence of various environmental and social sciences provides a new way of recognizing the connections and feedback between human and natural systems. To gain a better understanding of the interactions between humans and natural systems a socio-ecological framework has been introduced to integrate data from different natural and social science disciplines [22,23]. Meanwhile, the environment is increasingly being viewed and studied as socio-ecological system (SES) [24]. The SES is a complex dynamic system composed of humans and nature as two interacting parts, and the coupling relationship between human activities and the natural environment must be better understood [25,26,27]. Extensive comprehension of the potential for socio-ecological sustainability to change over time and the environment is critical to addressing this issue [23,28]. Identifying the evolutionary stages of the SES and the drivers in a long-term scale is critical for future system management [29]. Unfortunately, intertwined social-ecological research had typically focused on a single or small set of indictors of environmental effects. Yet, identification of the stage shifts of SES requires consideration of multiple indicators of affecting natural and social systems across space and time. 

Here we applied a comprehensive research framework to characterize how the interactive effects of drivers on karst SES during the millennium and their impact on local natural environment in Guizhou Province. We included five categories of main driver indicators (nature, society, climate, economics, and policy) in the analysis that affected the evolution of karst SES in Guizhou Province. We used historical documents and statistical data from Guizhou Province to quantify changes in indicators over the time and assess major impacts between different stages. We then attributed the expansion of rocky desertification to human-dominated land-use change in SES. Our research addresses the relative drivers and local impacts of karst SES over time on suite of indicators that can provide experience for future land use and related environmental management decisions in karst areas.

## 2. Materials and Methods

### 2.1. Study Area

**Figure 1:**
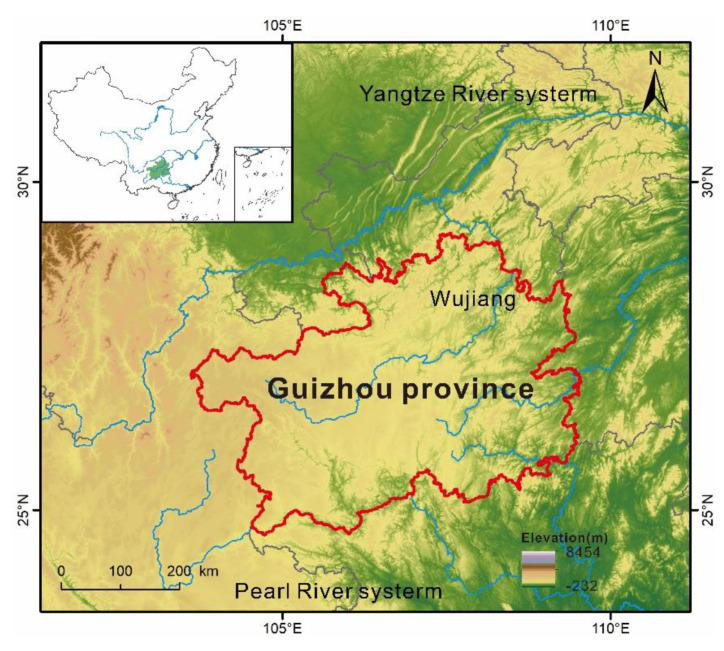
Location of the study area.

Guizhou Province is located in the eastern part of the Yunnan–Guizhou Plateau in Southwest China, longitudes of 103°36′–109°35′ E and latitudes of 24°37′–29°13′ N [30]. The terrain slopes gradually downward from west to east, spanning the Yangtze and Pearl Rivers (Figure 1). More than 90% of the land in this area is mountains or hills, with an average altitude of 1100 m [31,32]. This area has one of the highest concentrations of karsts in the world, with the largest continuous outcrop of carbonate rocks and densely developed karst [32]. The population pressure is enormous and many people live in poverty in remote mountain villages [31]. To produce enough food for subsistence, 80% of cultivated land is located in the hills and mountain areas with slope greater than 6°, resulting in high rock exposure rates, low-fertility, and shallow soil layers. The socioeconomic status of Guizhou is quite underdeveloped currently. 

### 2.2. Data Sources

The datasets used in this study are a combination of historical records, reconstructed data, and inferred data of published literature for historical periods, together with statistical data and experimental data for the period after 1949. We list the time span, spatial range, and source for each data in the datasets (Table 1). It is important to note that these datasets may have some errors and uncertainties. Due to the low temporal resolution and limitations of data accuracy and reliability, our study mainly reflects the general trends of these indicators over time without detailed analysis.

### 2.3. Framework for Identifying a Karst SES

**Figure 2:**
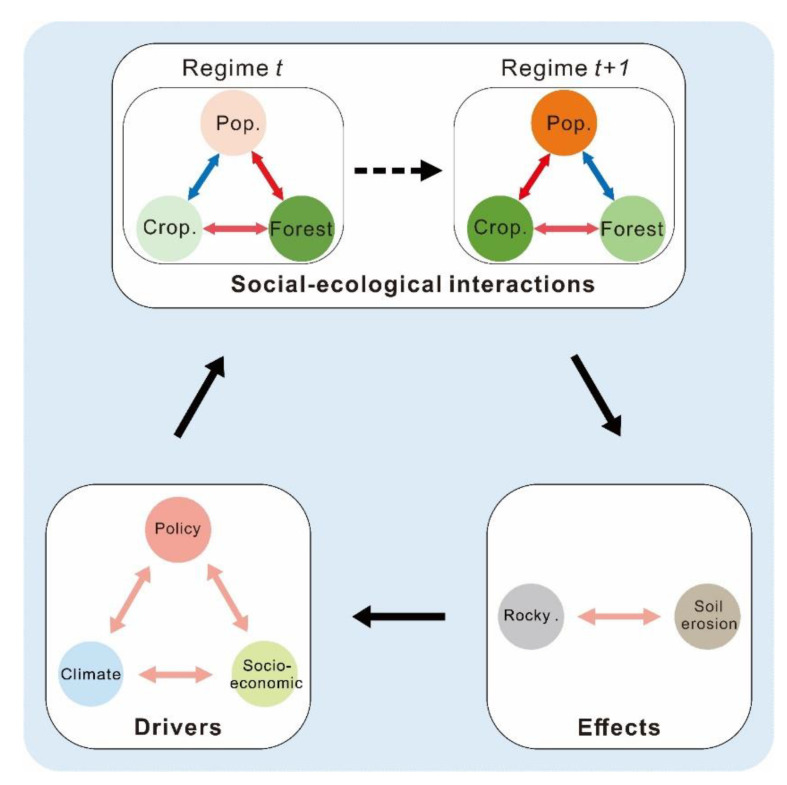
Framework for understanding the evolution of a karst SES.

We used a framework to empirically analyze the role of social processes on natural resource dynamics in Guizhou Province over the past 1000 years (Figure 2). The relationships between SES components were used to represent the interactions between system components and their transition. The interactions between system components remain unchanged in the static SES framework for each period. When the relationship of some SES components changes, it signifies an SES transition from one evolutionary period to another [53]. We divided each relationship into several periods by detecting the abrupt changes in the relationships between the system components based on piecewise linear regression (PLR). The evolutionary phases of the SES can be determined, and the evolution of SES can be analyzed by identifying the periods during which all the relationships remain unchanged.

Natural and social driving factors promote the evolution of the SES in Guizhou Province. In addition to the natural changes in the karst area, the long-term driving forces formed by the government policy, vicissitude of dynasties, social changes, and economic development affect the development process of Guizhou and directly or indirectly affect the local ecosystem. Population growth and related human behavior are keys to social development. Accordingly, population was chosen as an indicator for the social subsystem component. Forest coverage was selected as an indicator for the ecological subsystem component because forest coverage is closely related to ecological problems, such as soil erosion in karst areas. Cropland area, as a link between social and ecological subsystems through land use, was also selected as an indicator for the ecological subsystem component, which plays a crucial role in food security [53]. The interactions of these three indicators represent the relationship between population growth and the development of land-use change and a sustainable environment.

The other drivers that influence the system evolution are climate, policy, and social-economic influences. Climate drivers include temperature anomalies, a proxy precipitation index for historical periods, extreme drought, and flood events in Southwest China. Socioeconomic drivers include grain production per hectare, grain production, water conservancy projects, and war frequency. We also chose rocky desertification coverage as an indicator to evaluate the effects of social-ecological interactions in different regimes [54].

### 2.4. Identification of the Evolutionary Phases of a SES

We analyzed the nodes of mutation between SES components in the study time frame by using the PLR method. PLR is a statistical method that allows switching regressions to provide separate results for several segments of an independent variable [55]. The PLR was used to perform linear regression in two segments according to time. The boundary time between the segments is considered to be the turning point. PLR can be expressed as follows:

(1) $$Y=\{\begin{array}{c} a_{1}X+b_{1},\;T\leq T_{1}\\ a_{2}X+b_{2},T>T_{1} \end{array},$$

where *Y* is the dependent variable, *X* is independent variables, *a*_1_ and *a*_2_ are the slopes of the linear segments, *b*_1_ and *b*_2_ are the intercepts of linear segments, and *T*_1_ is the turning point. *T*_1_ was selected using two criteria: (i) time point with the least residual sum of squares of the regression lines and (ii) either a *p* value of the two regression lines before and after the breakpoint being less than 0.05. After the identification of a turning point, the other turning points of the segment (if they exist) were determined by the same method until no further time points met the criteria of turning point identification.

The study period was divided into several time segments by detecting split nodes that are abruptly changed between system components. These segments were then used to determine the evolutionary stage of the SES.

## 3. Results and Discussion

### 3.1. Division of the Evolutionary Phases of Karst SES

**Figure 3:**
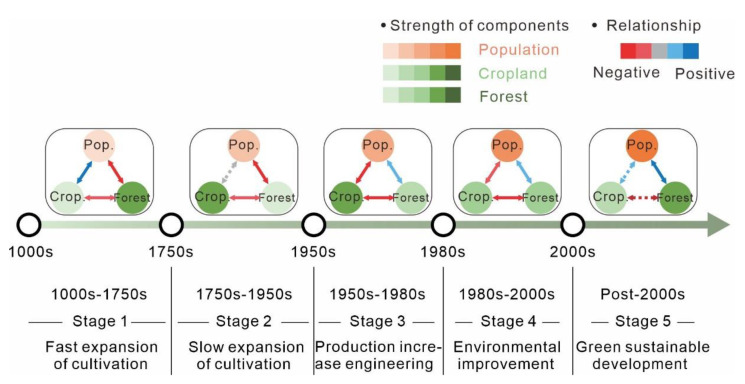
Evolutionary phases of the SES of Guizhou Province.

**Figure 4:**
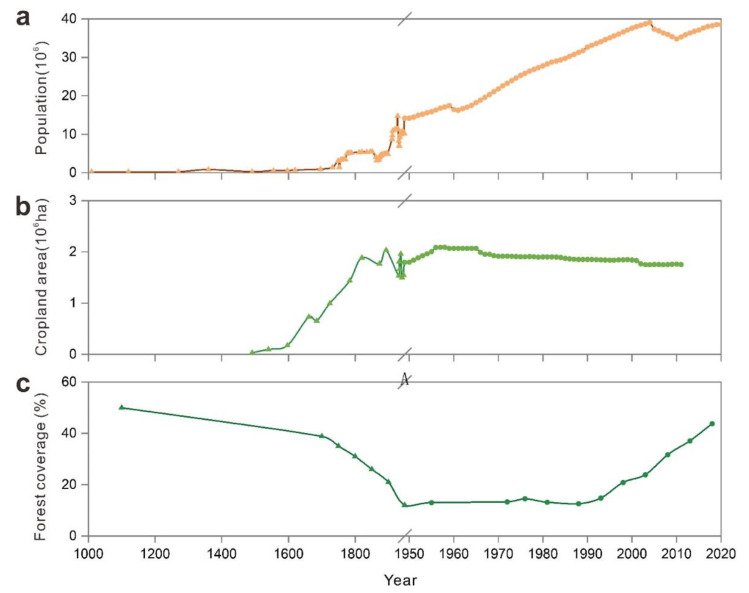
Changes in the SES component indicator over the past 1000 years: (**a**) population, (**b**) cropland area, and (**c**) forest coverage.

**Figure 5:**
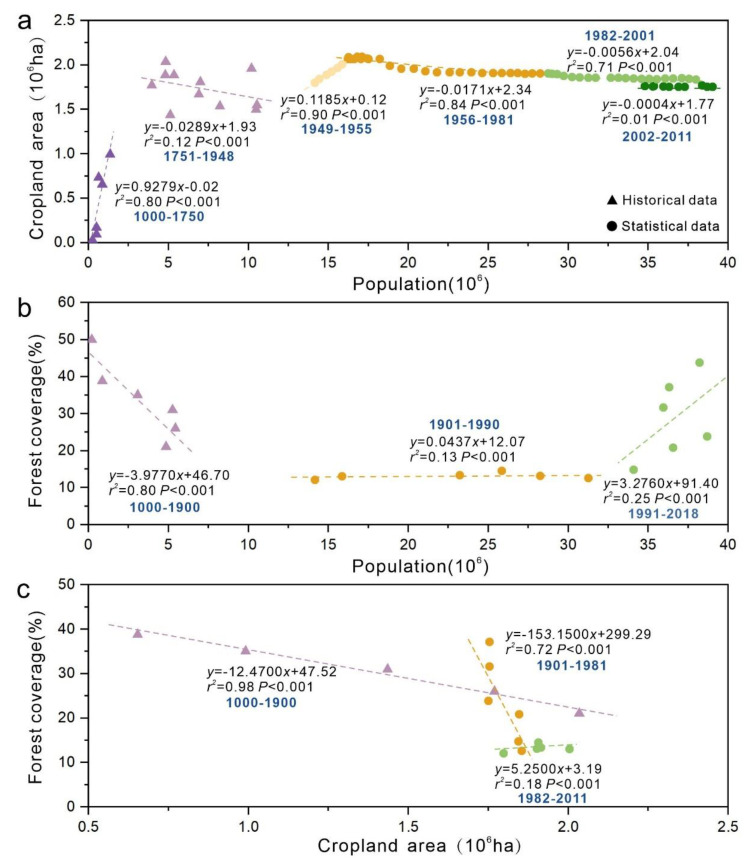
Changes in interactions between SES components of Guizhou Province. (**a**) Relationship between population and cropland area. (**b**) Relationship between population and forest coverage. (**c**) Relationship between cropland area and forest coverage.

We identified five evolutionary phases of SES in Guizhou Province over the past 1000 years (Figure 3) based on the changes in interactions between system components (Figure 4 and Figure 5):

The first phase (1000s–1750s) can be identified as ‘fast expansion of cultivation’. Population was positively correlated with cropland area and negatively correlated with forest coverage, and cropland area was negatively correlated with forest coverage. The positive regression slope of population and cropland area is relatively large, and the growth rate of cropland area is much larger than that of population change.

The second phase (1750s–1950s) can be identified as ‘slow expansion of cultivation’. The relationship between population and cropland area was not significant, and the slope of the regression line was negative. The negative regression slope of cropland and forest areas increased. Population and cropland area fluctuated during this period, and the forest coverage rate dropped to a minimum of 12% in 1949.

The third phase (1950s–1980s) can be described as ‘production increase engineering’. During this phase, population was negatively correlated with cropland area and positively correlated with forest coverage. The cropland area increased to 2.09 × 106 ha in the late 1950s and decreased to 1.90 × 106 ha in 1980, while population and forest cover steadily increased. The population more than doubled from 14.17 million in 1950 to 31.71 million in 1989. This period aimed to increase the productivity of cultivated land to feed the increasing population.

The fourth phase (1980s–2000s) can be characterized as ‘environmental improvement’. The negative regression slope of population and cultivated land area decreased, the population steadily increased, the cultivated land area continually decreased, and the forest coverage rate increased by about 21%.

The final phase (post-2000s) can be identified as ‘green sustainable development’. The relationship between population and cropland and between arable land and forest coverage became uncorrelated, whilst the positive regression slope between population and forest coverage increased. The population increased to 38.56 million. Meanwhile, cropland area decreased to about 1.75 × 106 ha, and forest coverage increased to about 44%.

### 3.2. Historical Overview of Evolution in Social-Ecological Interaction

#### 3.2.1. Fast Expansion of Cultivation: 1000s–1750s

Before the Ming Dynasty, most ethnic minorities in mountainous areas were less connected with the Central Plains, which led to their obsolete manufacturing techniques. In addition, the local Tusi chieftain’s rule and inconvenient transportation made it difficult for people to migrate from the Central Plains to Guizhou (Tusi is the leader of the minority tribes in southwest of ancient China) [19]. At that time, farming activities were mainly concentrated in the mountainous plain areas on a large scale. At this stage, ethnic minorities sustained the development of a society with a relatively low level of social development, a small scale of human settlement, and a limited scope of cultivated land. During the Yuan Dynasty (1271 AD–1368 AD), the government built a large number of official post roads, brought conveniences for the further development of Guizhou [19].

**Figure 6:**
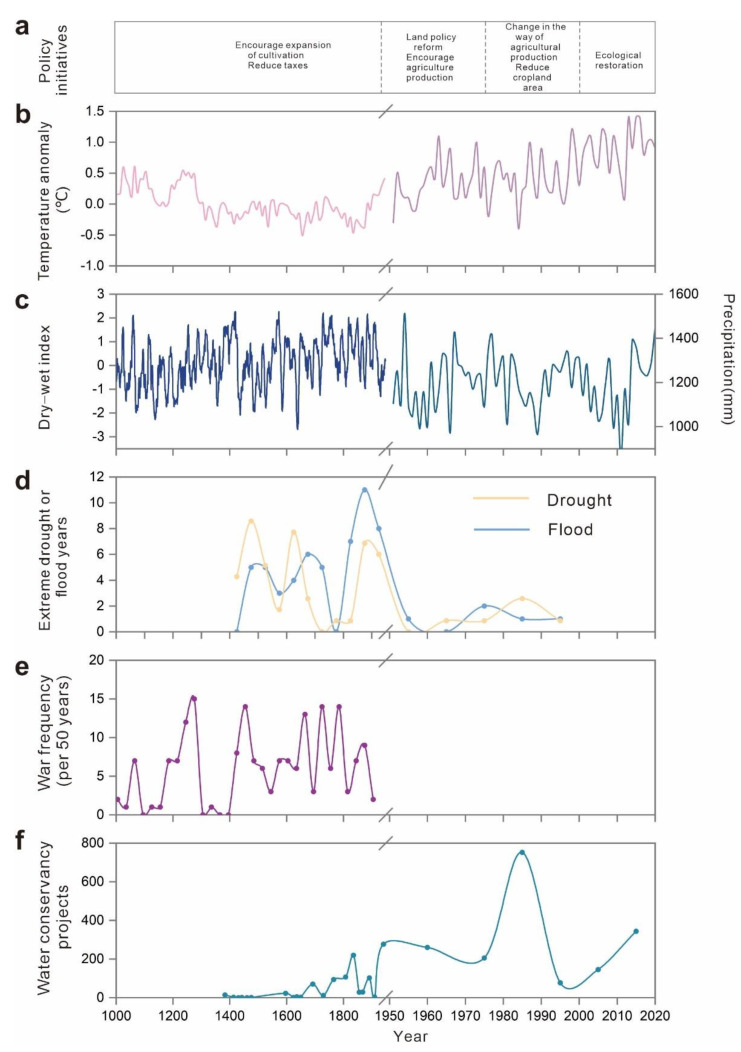
Drivers of changes in socio-ecological interactions of Guizhou Province. (**a**) Policy initiatives, (**b**) temperature anomaly, (**c**) dry-wet index, (**d**) extreme drought or flood years, (**e**) war frequency, (**f**) water conservancy projects.

**Figure 7:**
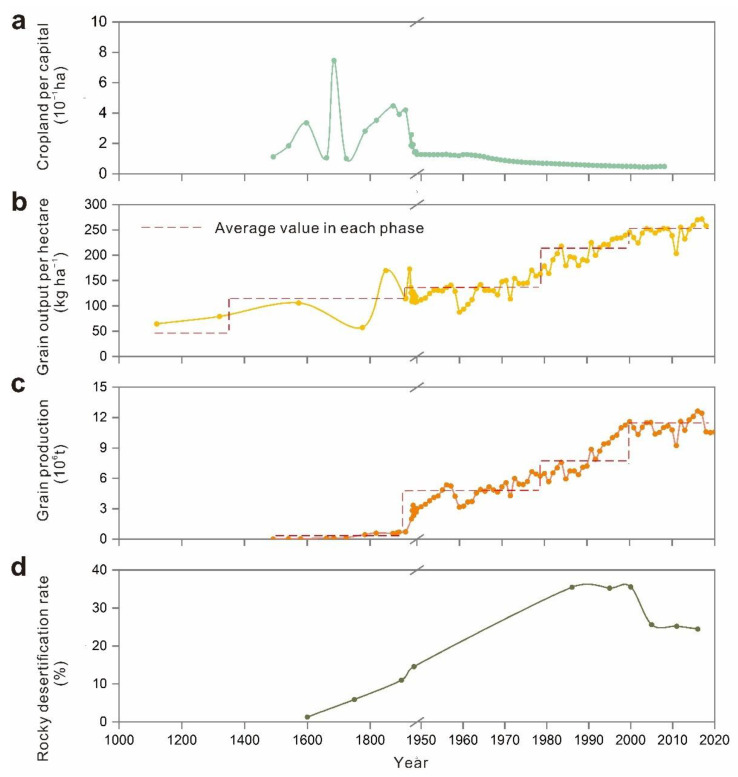
Effects of change in socio-ecological interactions on (**a**) cropland area per capital, (**b**) grain output per hectare, (**c**) grain production, and (**d**) rocky desertification rate.

The Ming Dynasty established the provincial system in Guizhou around 1413 AD, which implemented the policy of ‘Replacement of the Tusi Chieftain with Government-dispatched Officials’ and directly managed by the central government [56]. This policy initially opened up the original closed development environment in Guizhou. With the large number of immigrants from the Central Plains, agricultural production in low-altitude areas, such as riparian areas with better natural conditions, developed rapidly due to convenient irrigation conditions [57]. The agricultural production technology brought by the immigrants in the Central Plains had been disseminated and promoted. In particular, the cattle farming technique had been paid increasing attention, which directly changed the backward agricultural production situation in the minority areas of Guizhou. The government enhanced the enthusiasm of armers for developing or reclaiming new cropland by providing preferential treatment for farmers and reducing corvée tax. More advanced agricultural productivity was achieved by constructing hydraulic facilities and improving irrigation techniques [44]. Farmers began to reclaim new land on the edge of the hillside with a relatively gentle slope when the fertility of the soil on flat land declined due to extensive farming expansion. At the end of the Ming Dynasty, many farmlands were abandoned due to years of wars (Figure 6e), land-use change activities had been severely affected, and the growth rate of cultivated land began to decline. Accordingly, the Qing government had carried out a series of reforms of land tax systems and corvée tax policies to restore agricultural production. In particular, the emperor Kangxi of the Qing Dynasty decided to freeze taxes on corvée labor of various local governments instead of imposing additional taxes on corvée labor as the population increased, which greatly promoted the growth of population and cropland [36]. During this period, high-yielding crops (e.g., corn, potato, and sweet potato) were introduced to Guizhou from the Americas, and they could grow on poor land with steep slope. As a consequence, agriculture flourished in the mountains, with continuous deforestation to grow crops on the slopes. In addition, during this cold climate period the drop in temperature reduces heat input (Figure 6b), resulting in lower food production per unit area (Figure 7b). Moreover, the increased duration of droughts and floods (Figure 6d) also hindered agricultural production at that time. Consequently, plenty of nature vegetations needed to be cleared to feed the population at that time.

In summary, the expansion of land use was promoted by factors such as the population growth caused by the construction of roads and the migration of residents from the Central Plains, the advanced production technology, the reduction in taxes and the introduction of high-yield foreign crops. In addition, the decrease in grain production caused by the drop in temperature also contributed to the expansion of cropland. Unfortunately, the war led to the abandonment of cropland.

#### 3.2.2. Slow Expansion of Cultivation: 1750s–1950s

During this period, the scope of human activities continued to expand with the acceleration of population growth, and the area of cultivated land also continued to expand. In the middle and late Qing Dynasty, the cultivation of poppies occupied a large amount of high-quality cultivated land, and agricultural production was greatly damaged. People chose to cultivate more sloping cultivated land to support their lives. In the late Qing Dynasty and the period of the Republic of China, land-use change was slowed down by the frequent wars (Figure 6e). The war also stagnated the construction of water conservancy projects (Figure 6f), which was not conducive to agricultural production. In the meantime, a large number of people from the China Central Plains migrated to Guizhou. The government called on these people to expand land-use change to solve the problem of food demand. 

Poppy occupation of low-lying farmland led to the expansion of arable land into hillsides. The war led to the decline of population, the abandonment of arable land, and the decline or even stagnation of the expansion of arable land.

#### 3.2.3. Production Increase Engineering: 1950s–1980s

In the 1950s, the stable domestic situation in China led to a sharp increase in the population and the orderly agricultural production activities. Arable land-use change activities were stimulated by the grain-oriented policy, and sloping land-use substantially increased. During the ‘Great Leap Forward’, almost all forests except protected areas were wiped out. The area of cultivated land reached its maximum value during this period. At this time, the attitudes of the government and residents towards to the environment dramatically changed due to the outbreak of environmental problems. To effectively reduce local ecological environmental degradation, terraces and walls were constructed on hill slopes. Berms and earth dams were used to retain moisture in cultivated land and raise the water level in creeks for irrigation [9]. The agricultural productivity increased owing to the improvements in farming practices and construction of farmland projects (Figure 7b). 

The stable political environment led to population explosion. In order to cope with the increasing demand for food, farmland was further expanded and environmental problems broke out. The government has introduced a series of grain-oriented policies, while the improvement of farming practices and the construction of farmland projects have also played a certain role in solving the food problem and alleviating environmental degradation.

#### 3.2.4. Environmental Improvement: 1980s–2000s

The economy of China rapidly developed following the reform and opening-up policy in the late 1970s [58]. Under the political and economic pressures, farmers were encouraged to change extensive land-use activities to more intensive production practices, or to other land-use practices designed for protecting biodiversity and ecosystem services [59]. Moreover, government and farmers strengthened the scientific and technological investment in agricultural production and irrigation facilities (Figure 6f). As a result, the grain yield per unit area significantly increased, which maintained larger populations on virtually the same area [58]. In order to control soil erosion, improved cropland productivity, and gradually establishing a benign agricultural ecosystem, the policy ‘comprehensive management of small watershed’ was launched to establish a three-level comprehensive protection system for slopes, channels, and basic cropland [60]. During this period, the speed of agricultural land-use change slowed down, and the agricultural production mode gradually shifted from expanding cropland to increasing productivity. Although the population continued growing (Figure 4a), the pressure of the population on cropland was gradually reduced.

In brief, with the development of economy, agriculture entered the intensive mode, agricultural production input increased, and agricultural production level further enhanced. At the same time, land-use change has slowed down and ecological protection has strengthened.

#### 3.2.5. Green Sustainable Development: Post-2000s

At the end of the 20th century, the central government of China launched a series of large-scale ecological restoration programs to restore ecosystem services and ensure sustainable development [61,62]. To protect existing natural forests, ‘Natural Forest Protection Program’ (NFPP) was initiated in 1998 by closing mountains and afforestation. In 1999, ‘Grain to Green’ policy (GTGP) was launched by returning farmland to forest and grassland to mitigate forest damage [63]. Moreover, in order to reduce natural disasters, the ‘Slope Land Conversion Project’ (SLCP) was launched to convert cultivated land on slopes greater than 25° into forest land. Moreover, to alleviate the food security issue owing to the continuous reduction in cropland, the ‘Regulation of Basic Farmland Protection’ ensured scarce arable land resource [58]. The reduction in arable land also freed up rural labor from crop production and facilitated a shift to off-farm activities. Based on the above programs and outcomes, agricultural land-use change activities ceased and the area of arable land began to decrease during this period.

In summary, a large-scale ecological restoration program was launched, and steep slope arable land was converted to forest and grassland, making the ecological environment more sustainable.

### 3.3. Direct and Latent Influences of Social-Ecological Interactions 

In different stages, socio-ecosystem drivers interact with each other to have direct or indirect impacts on the local environment, some of which persist into modern times and produce new impact outcomes. In the early stages, agricultural production was the main driving force for social development. To increase the development of Guizhou, the government encouraged the land-use change in arable land and expanded the cultivation area, meeting the needs of population growth for food and ignoring the vulnerability of the local ecological environment. The land-use change in sloping land was more likely to cause soil erosion, and the occurrence area of rocky desertification was also dominated by steep slopes [19]. The unreasonable agricultural practice had caused soil loss and rocky desertification began to appear [64]. Moreover, the introduction of high-yielding crops from the Americas had plunged this region into a vicious cycle. These crops increased food production and fed a larger population, which stimulated people to reclaim more land (especially sloping land) and deforestation [65]. Although agricultural productivity was greatly improved, rocky desertification spread further [19]. In the middle of the Qing Dynasty, the forest coverage of Guizhou dropped to about 30% (Figure 4c). The outbreak of war left a lot of arable land abandoned, and the wasteland without vegetation cover was prone to soil loss. Deforestation and wasteland development in Guizhou were more widespread during the Republic of China period. Underground runoff was reduced and soil erosion was serious because a large number of forests were cut down. As a result, the advent of heavy rainfall could easily lead to flash floods and frequent disasters. In addition, soil erosion also increased the degree of rocky desertification [66]. The dramatic increase in population forced people to cultivate on more sloping lands and constantly clear the forest and shrubs since the 1950s [9,67]. In particular, the ‘Great Leap Forward’ campaign wiped out almost all forests except for a few reserves [9], and the forest coverage rate reached its lowest value during this period (Figure 4c). The decline of vegetation coverage accelerated soil erosion, which in turn induced the expansion of rocky desertification (Figure 7d). In recent decades, the government implemented policies and projects aimed at maintaining and restoring ecosystem services in karst areas, reducing soil erosion, and rocky desertification. Unfortunately, those projects did not take into account the importance of local species diversity and landscape patterns. Whether the current implementation of these projects is sustainable remains to be verified. For example, despite the increase in forest cover in some areas, the replacement of native forests by monoculture planted forests has reduced the proportion of native forests. Especially, forests converted from cultivated land, which has reduced biodiversity [68] and influenced infiltration, erosion, and organic matter inputs [69]. In addition, the planting of some fast-growing tree species has a negative impact on soil quality, resulting in the decline of soil moisture and nutrients, breaking the balance of water supply and demand in the region [70]. The planting of large commercial forests increases evapotranspiration and has implications for water use efficiency [71]. Artificial chemical fertilizers were used by farmers to improve the productivity of their arable land, but agricultural pollution from fertilizers, pesticides, and herbicides threatens vegetation and crops, which alters the water cycle by affecting the balance and transformation of soil moisture and nutrients [72].

### 3.4. Analysis of Historical Trend in Land Use and Rocky Desertification 

**Figure 8:**
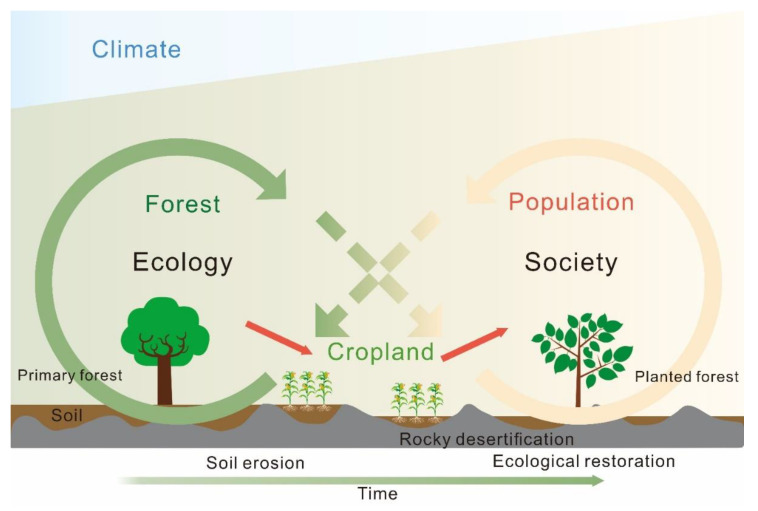
Conceptual evolution of a karst SES with increasing population pressure and human impacts.

The millennial historical evolution of Guizhou Province reflects the changes in karst land-use patterns. All kinds of natural factors and social factors of SES have a direct or indirect relationship with land use, some of them can drive land-use change, and some of them become the spillover effect of land-use change (Figure 8). Moreover, some drivers have significant changes at different stages [53]. Rocky desertification caused by karst ecological degradation is one of the most significant spillover effects of land-use change. Our study reveals land-use change because unregulated agricultural expansion has led to exacerbation of rocky desertification, which aligns with other research [18,19]. We systematically analyzed the evolution of the karst SES through a temporal perspective to reveal the relationship between land use and rocky desertification. In the early stages (stage 1, 2 and 3), human activities focused on achieving food security. Local people continued to expand agriculture in order to feed the growing population, coupled with continued government measures to encourage planting, the scope of agricultural reclamation continues to expand. During those periods, agricultural development was mainly achieved by changing natural landscapes [66], resulting in degradation of local environment. In particular, people began to clear and cultivate on a large number of steep slopes with forests when the arable land in flat areas was lacking. Long-term cultivation on a hillside with a thin soil layer caused soil erosion, and land degradation to occur [73], and the rocky desertification was aggravated. Accordingly, with the intensification of the contradiction between people and land, the ecological environment was destroyed dramatically. This created a vicious cycle of overplanting and environmental degeneration. The destruction of the ecological environment reduced land productivity and food production capacity, undermining long-term food security. Furthermore, the falling temperature, droughts, or floods caused famines and social unrest, which eventually triggered wars. In recent decades, people have become increasingly aware of the importance of ecological protection, and the government has implemented many policies to protect environment. Since the implementation of ‘Grain for Green’ program in 1999 [63], the cropland area on hillslopes in Guizhou province has decreased substantially by the conversion to forests and grasslands. Correspondingly, the rocky desertification area began to decline (Figure 7d).

On the whole, the transition from pursuing food production whilst ignoring environmental degradation to being environmentally sustainable is a slow process that takes hundreds of years. This transition did not happen until there was direct evidence that unreasonable agricultural land-use change and farming practices had exacerbated ecological degradation in karst areas. Government policy plays a key role in this transition, and environmental action changes more rapidly after policy implementation. 

### 3.5. The Significance of Studying the Millennial Evolution of Karst

We examined the millennial historical evolution of Guizhou Province through the perspectives of interactive processes between a karst SES components. Five stages of evolution were identified as fast expansion of cultivation, slow expansion of cultivation, production increase engineering, environmental improvement, and green sustainable development. Unlike many studies that describe karst evolution from a simple historical perspective, we describe it based on ecology as the theoretical basis and combine social, economic, political, and other factors. Natural, social, and political factors interact and jointly drive the evolution of karst SES. Our study links the historical stages of SES with their driving factors and influences, which facilitates our in-depth analysis of the karst historical evolution. In different periods, the proportion of natural, political, and social driving factors determines which state SES should reach. In general, the proportion of natural drivers gradually decreases, while the proportion of social and political drivers gradually increases, and political driving starts to dominate. Land-use change under human activities affects and changes the structure and function of karst ecosystem, and thus has great impact on human society. This study is more detailed than previous studies, which makes it interpretive and predictive. Furthermore, this study, as one of the research across social and natural sciences, provides a new idea for future study on karst regions.

We described the evolution of a karst SES over a thousand years and identified its transformation patterns over time. The qualitative and quantitative method used in this work may make this study interpretable and predictable. However, long-term research on the evolution of SES lack literature, especially the data about historical periods, making it difficult to integrate data from different historical periods and quantify system components. Accordingly, the historical evolution of the karst SES determined in this study is only a rough description of the historical reality. This study only reflects the overall historical situation of Guizhou through the study of its data sources, while the actual situation in some areas may be slightly different from the results of this study. Such as previous studies on millennial evolution of socio-ecological systems [53,74], our study was also based on large SES-managed region with long histories and cross-scale effects. Compared with the Loess Plateau and Inner Mongolia, the karst region of Southwest China is unique in its geological structure and environmental problem (i.e., rocky desertification). However, the research on the karst socio-ecological system has not been reported in currently published papers. This study can facilitate filling this gap and reveal the millennial-scale evolution of a karst socio-ecological system and its implication for environmental management concerning combating rocky desertification. In addition, we found that these large SES management areas were currently under more pressure from food and other resource demand than in any historical period. Managers need to strengthen supervision and implement existing management policies to protect ecological resources and improve people’s well-being.

The expansion of rocky desertification in Guizhou has been accelerated in the past thousand years due to the special karst geological conditions and the destruction of unreasonable human activities in history. Although Guizhou has made great achievements in the control of rocky desertification and ecological restoration in recent decades, sustainable development will face huge challenges in the future. Our work provides an understanding of the evolution of karst land use and rocky desertification from the interaction of social systems and ecosystems. This study can help managers and policymakers identify future practices that need to be improved by understanding how ecological problems in today’s karst were created over time in history.

## 4. Conclusions

In this study, our quantitative analysis of the spatio-temporal dynamics of evolution of karst SES during the millennium demonstrates that movement towards a more sustainable ecological environment is possible, and indeed is occurring. Meanwhile, land-use change to human activities is the leading driver of overall trends of expansion of rocky desertification. Moreover, the change in land use and the evolution process of rocky desertification have been synchronously carried out. The change in land resource utilization brought by different periods of agricultural development or the political decision of the government has a great impact on Guizhou’s ecosystem. Some of these impacts affect the social system and the ecological environment, whilst others directly change the local ecological structure and landscape. However, the improvement of agricultural production efficiency can alleviate the increasing pressure of human activities on environment. To achieve a sustainable karst ecological environment, the coupling of human activities with the natural environment must be noted. Taking advantage of the potential to enhance local ecosystem service function while offsetting negative impacts of historical human activities is critical to environmentally sustainable development. Therefore, our outcomes that trace the causes of current ecological and environmental problems in Southwest China’s karst regions are meaningful. Guizhou is facing many issues, such as ecological environment degradation, food security, and the future direction of sustainable development. Our research provides some lessons for managers and policymakers. This study can assist managers and policymakers to identify how environmental issues emerged of karst areas and how they should be addressed.

## Figures and Tables

**Table 1:** Information about the datasets.

Dataset	Type	Time Span	Spatial Range	Data Source
Population	Historical records, inferred data, statistical data	1000–2020	Guizhou Province	[33,34,35], National Bureau of Statistics of China (www.stats.gov.cn (accessed on 20 April 2022))
Cropland area	Historical records, reconstructed data, statistical data	1500–2011	Guizhou Province	[35,36,37,38]
Forest coverage	Reconstructed data inferred data, statistical data	1100–2018	Guizhou Province	[39,40], National Forest Resource Surveys (www.forestry.gov.cn (accessed on 20 April 2022))
Cropland per capital ^1^	Inferred data	1500–2011	Guizhou Province	-
Grain yield ^2^	Inferred data, statistical data	1100–2018	Guizhou Province	[35,41,42,43]
Grain production ^3^	Historical records, inferred data, statistical data	1500–2020	Guizhou Province	[33,35]
Water conservancy projects ^4^	Historical records, statistical data	1380–2015	Guizhou Province	[44,45,46], National Bureau of Statistics of China (www.stats.gov.cn (accessed on 20 April 2022))
Extreme drought and flood events	Reconstructed data	1400–2000	Southwest China	[47]
War frequency	Historical records	1000–1949	Southwest China	[48,49]
Rocky desertification rate	Experimental data, inferred data	1600–2016	Guizhou Province	[19,50], China Karst Bulletin
Temperature anomaly	Reconstructed data	1000–1949	Eastern China	[51]
Proxy precipitation index	Reconstructed data	1000–1949	Central and eastern China	[52]
Temperature	Statistical data	1951–2020	Guizhou Province	[35], Guizhou Bureau of Statistics (www.stats.gov.cn (accessed on 20 April 2022))
Precipitation	Statistical data	1951–2020	Guizhou Province	[35], Guizhou Bureau of Statistics (www.stats.gov.cn (accessed on 20 April 2022))

^1^ The data of cropland per capital are calculated from data of population and cropland area. ^2^ Grain yield data of the Republic of China were calculated from data of grain production and cropland area. ^3^ Grain yield data of the period before the Republic of China were calculated from data of grain yield and cropland area. ^4^ The data of water conservancy projects after 1949 were represented by the number of dams constructed for convenience of connection with historical data.

## Data Availability

All data needed to evaluate the conclusions in the paper are present in the paper. Additional data related to this paper may be requested from the authors.

## References

[1] Foley J.A., DeFries R., Asner G.P., Barford C., Bonan G., Carpenter S.R., Chapin F.S., Coe M.T., Daily G.C., Gibbs H.K. (2005). Global consequences of land use. Science.

[2] Vitousek P.M., Mooney H.A., Lubchenco J., Melillo J.M. (1997). Human domination of Earth’s ecosystems. Science.

[3] Board M.A. (2005). Millennium Ecosystem Assessment.

[4] Council N.R. (2001). Grand Challenges in Environmental Sciences.

[5] Omenn G.S. (2006). Grand challenges and great opportunities in science, technology, and public policy. Science.

[6] Morrison K.D., Tello E., Hammer E., Popova L., Madella M., Whitehouse N., Gaillard M.-J. (2018). Global-scale comparisons of human land use: Developing shared terminology for land-use practices for global change. Past Glob. Chang. Mag..

[7] Fang X., Ye Y., Zhang C., Tang C. (2019). Cropland cover change and its environmental impacts in the history of China. J. Palaeogeogr..

[8] Bai X., Wang S., Xiong K. (2013). Assessing spatial-temporal evolution processes of karst rocky desertification land: Indications for restoration strategies. Land Degrad. Dev..

[9] Jiang Z., Lian Y., Qin X. (2014). Rocky desertification in Southwest China: Impacts, causes, and restoration. Earth -Sci. Rev..

[10] Yuan D. (1997). Rock desertification in the subtropical karst of south China. Z. Für Geomorphol..

[11] Wu L., Wang S., Bai X., Luo W., Tian Y., Zeng C., Luo G., He S. (2017). Quantitative assessment of the impacts of climate change and human activities on runoff change in a typical karst watershed, SW China. Sci. Total Environ..

[12] Ouyang Z., Song T., Peng W., Zeng F. (2011). Spatial heterogeneity of soil main mineral composition in manmade forest in karst peak-cluster-depression region. J. Hunan Agric. Univ..

[13] Zeng C., Wang S., Bai X., Li Y., Tian Y., Li Y., Wu L., Luo G. (2017). Soil erosion evolution and spatial correlation analysis in a typical karst geomorphology using RUSLE with GIS. Solid Earth.

[14] Xiong K., Chi Y. (2015). The problems in southern China karst ecosystem in southern of China and its countermeasures. Ecol. Econ..

[15] Hu Z., Wang S., Bai X., Luo G., Li Q., Wu L., Yang Y., Tian S., Li C., Deng Y. (2020). Changes in ecosystem service values in karst areas of China. Agric. Ecosyst. Environ..

[16] Zhang G., Zhu Y., Shao M.A. (2019). Understanding sustainability of soil and water resources in a critical zone perspective. Sci. China Earth Sci..

[17] Liu Z., Sun H., Li H., Wan N. (2011). δ13C, δ18O and deposition rate of tufa in Xiangshui River, SW China: Implications for land-cover change caused by climate and human impact during the late Holocene. Geol. Soc. Lond. Spec. Publ..

[18] Chen C., Yuan D., Cheng H., Yu T., Shen C., Edwards R.L., Wu Y., Xiao S., Zhang J., Wang T. (2021). Human activity and climate change triggered the expansion of rocky desertification in the karst areas of Southwestern China. Sci. China Earth Sci..

[19] Cheng A., Wang S., LI B., Bai X., Ni X. (2010). Evolution History of Karst Rocky Desertification and Its Significance in Guizhou Province. Bull. Soil Water Conserv..

[20] Zhou Y. (2006). The Historic Progress and Sustained Development of Man-Earth Relationship of GuiZhou Province. J. Taiyuan Norm. Univ..

[21] Zou Y. (2009). Historical development and implication of agriculture in Guizhou province. Tillage Cultiv..

[22] Leslie H.M., Basurto X., Nenadovic M., Sievanen L., Cavanaugh K.C., Cota-Nieto J.J., Erisman B.E., Finkbeiner E., Hinojosa-Arango G., Moreno-Báez M. (2015). Operationalizing the social-ecological systems framework to assess sustainability. Proc. Natl. Acad. Sci. USA.

[23] Liu J., Dietz T., Carpenter S.R., Alberti M., Folke C., Moran E., Pell A.N., Deadman P., Kratz T., Lubchenco J. (2007). Complexity of coupled human and natural systems. Science.

[24] Collins S.L., Carpenter S.R., Swinton S.M., Orenstein D.E., Childers D.L., Gragson T.L., Grimm N.B., Grove J.M., Harlan S.L., Kaye J.P. (2011). An integrated conceptual framework for long-term social–ecological research. Front. Ecol. Environ..

[25] Ostrom E. (2009). A general framework for analyzing sustainability of social-ecological systems. Science.

[26] Liu J., Mooney H., Hull V., Davis S.J., Gaskell J., Hertel T., Lubchenco J., Seto K.C., Gleick P., Kremen C. (2015). Systems integration for global sustainability. Science.

[27] Bao W., Gong A., Zhao Y., Chen S., Ba W., He Y. (2022). High-Precision Population Spatialization in Metropolises Based on Ensemble Learning: A Case Study of Beijing, China. Remote Sens..

[28] Levin S.A., Clark W. (2010). Toward a science of sustainability: Report from toward a science of sustainability conference. CID Work. Pap. Ser..

[29] Reyers B., Folke C., Moore M.-L., Biggs R., Galaz V. (2018). Social-ecological systems insights for navigating the dynamics of the Anthropocene. Annu. Rev. Environ. Resour..

[30] Liu L. (2021). Assessment of water resource security in karst area of Guizhou Province, China. Sci. Rep..

[31] Qiu S., Peng J., Dong J., Wang X., Ding Z., Zhang H., Mao Q., Liu H., Quine T.A., Meersmans J. (2021). Understanding the relationships between ecosystem services and associated social-ecological drivers in a karst region: A case study of Guizhou Province, China. Prog. Phys. Geogr. Earth Environ..

[32] Qian C., Qiang H., Zhang G., Li M. (2021). Long-term changes of forest biomass and its driving factors in karst area, Guizhou, China. Int. J. Distrib. Sens. Netw..

[33] Li D., Jiang D. (1987). Selected Materials of Modern Economic History of Guizhou (Part 1).

[34] Lv Z. (1999). China Guizhou Population Research.

[35] Guizhou Provincial Bureau of Statistics (2019). 70 Years in Guizhou (1949–2019).

[36] Ge Q., Dai J., He F., Zheng J., Man Z., Zhao Y. (2004). Spatiotemporal dynamics of reclamation and cultivation and its driving factors in parts of China during the last three centuries. Prog. Nat. Sci..

[37] Yang S. (2010). Restoration of the distribution of rocky desertification in Guizhou during the Republic of China and its causes.

[38] Zhao P., Wu J. (2021). The Annals of Guizhou in the Wanli Period (Calibration Version).

[39] He F., Ge Q., Dai J., Rao Y. (2008). Forest change of China in recent 300 years. J. Geogr. Sci..

[40] Lan Y. (1992). Economic Development and Ecological Changes of Southwest China in History.

[41] Chen G. (1994). A Small Study on the Yield of Rice Per Mu in Guizhou during the Wanli Period. J. Chin. Hist. Geogr..

[42] Chen X. (1995). An Analysis of Grain Yield per Mu in Yuan Dynasty. Hist. Res..

[43] Perkins D.H. (2013). Agricultural Development in China, 1368–1968.

[44] Xu N., Zhang Y. (2017). Spatiotemporal distribution and regional characteristics of water conservancy in Guizhou in Ming and Qing Dynasties. Theory Res..

[45] Wan Q. (2021). GuiZhou University of Finance and Economics.

[46] Guizhou Province Local Chronicle Compilation Committee (1997). Chorography of Guizhou Province -Water Conservancy.

[47] Liu W., Yang Y. (2021). Reconstruction and analysis of extreme drought and flood events in Southwest China in the past 600 years. Quat. Sci..

[48] Wang J. (2007). Research of the Relationship between Climatic Changes and Wars in Chinese History. Master’s Thesis.

[49] Editorial Board of Chinese Military History (1983). Chinese Military History.

[50] Bai X., Wang S., Chen Q., Cheng A., Ni X. (2009). Spatio-temporal evolution process and its evaluation method of karst rocky desertification in Guizhou Province. Acta Geogr. Sin..

[51] Ge Q., Hao Z., Zheng J., Shao X. (2013). Temperature changes over the past 2000 yr in China and comparison with the Northern Hemisphere. Clim. Past.

[52] Zheng J., Wang W., Ge Q., Man Z., Zhang P. (2006). Precipitation variability and extreme events in eastern China during the past 1500 years. TAO Terr. Atmos. Ocean. Sci..

[53] Wu X., Wei Y., Fu B., Wang S., Zhao Y., Moran E.F. (2020). Evolution and effects of the social-ecological system over a millennium in China’s Loess Plateau. Sci. Adv..

[54] Yu P., Wang Y., Coles N., Xiong W., Xu L. (2015). Simulation of runoff changes caused by cropland to forest conversion in the upper Yangtze River region, SW China. PLoS ONE.

[55] Malash G.F., El-Khaiary M.I. (2010). Piecewise linear regression: A statistical method for the analysis of experimental adsorption data by the intraparticle-diffusion models. Chem. Eng. J..

[56] Luo Q. (2019). On the Military Garrison and Defense System of Guizhou during the Ming Dynasty. China’s Borderl. Hist. Geogr. Stud..

[57] Li J. (2014). Immigrants and agricultural development of Guizhou province in Ming and Qing dynasties. Agric. Archaeol..

[58] Peng J., Xu Y., Cai Y., Xiao H. (2011). The role of policies in land use/cover change since the 1970s in ecologically fragile karst areas of Southwest China: A case study on the Maotiaohe watershed. Environ. Sci. Policy.

[59] van Vliet N., Mertz O., Heinimann A., Langanke T., Pascual U., Schmook B., Adams C., Schmidt-Vogt D., Messerli P., Leisz S. (2012). Trends, drivers and impacts of changes in swidden cultivation in tropical forest-agriculture frontiers: A global assessment. Glob. Environ. Chang..

[60] Chen W. (1999). Preliminary solution for food problem of small watershed in carst areas of Guizhou Province. Bull. Soil Water Conserv..

[61] Bryan B.A., Gao L., Ye Y., Sun X., Connor J.D., Crossman N.D., Stafford-Smith M., Wu J., He C., Yu D. (2018). China’s response to a national land-system sustainability emergency. Nature.

[62] Schmidt-Traub G., Locke H., Gao J., Ouyang Z., Adams J., Li L., Sala E., Shaw M.R., Troëng S., Xu J. (2021). Integrating climate, biodiversity, and sustainable land-use strategies: Innovations from China. Natl. Sci. Rev..

[63] Yeh E.T. (2009). Greening western China: A critical view. Geoforum.

[64] Liu Z., Wang H., Zhang Q. (2014). The Research for Historical Events of Development of Rocky Desertification Based on Speleothems Records. Sci. Technol. Eng..

[65] Ma C., Han Z. (2019). A preliminary study on the distribution and causes of rocky desertification in Yunnan during the Ming and Qing dynasties. J. Yunnan Univ..

[66] Cao Z., Ke Q., Zhang K., Zhang Z., Liu Y., Xiao S., Wei M. (2022). Millennial scale erosion and sedimentation investigation in karst watersheds using dating and palynology. Catena.

[67] Qiu S., Peng J., Zheng H., Xu Z., Meersmans J. (2022). How can massive ecological restoration programs interplay with social-ecological systems? A review of research in the South China karst region. Sci. Total Environ..

[68] Hua F., Wang L., Fisher B., Zheng X., Wang X., Douglas W.Y., Tang Y., Zhu J., Wilcove D.S. (2018). Tree plantations displacing native forests: The nature and drivers of apparent forest recovery on former croplands in Southwestern China from 2000 to 2015. Biol. Conserv..

[69] Ramankutty N., Mehrabi Z., Waha K., Jarvis L., Kremen C., Herrero M., Rieseberg L.H. (2018). Trends in global agricultural land use: Implications for environmental health and food security. Annu. Rev. Plant Biol..

[70] Yu F., Huang X., Liang Q., Yao P., Li X., Liao Z., Duan C., Zhang G., Shao H. (2015). Ecological water demand of regional vegetation: The example of the 2010 severe drought in Southwest China. Plant Biosyst. Int. J. Deal. All Asp. Plant Biol..

[71] Tong X., Brandt M., Yue Y., Ciais P., Rudbeck Jepsen M., Penuelas J., Wigneron J.-P., Xiao X., Song X.-P., Horion S. (2020). Forest management in southern China generates short term extensive carbon sequestration. Nat. Commun..

[72] Falloon P., Betts R. (2010). Climate impacts on European agriculture and water management in the context of adaptation and mitigation--the importance of an integrated approach. Sci. Total Environ..

[73] Zhang D., Ouyang Z., Wang S. (2001). Population resources environment and sustainable development in the karst region of southwest China. China Popul. Resour. Environ..

[74] Robinson B.E., Li P., Hou X. (2017). Institutional change in social-ecological systems: The evolution of grassland management in Inner Mongolia. Glob. Environ. Chang..

